# Reducing Waiting Times and Admission Periods through Pre-Admission Testing: A Quality Improvement Study on In-Hospital Renal Biopsy

**DOI:** 10.3390/jcm13123445

**Published:** 2024-06-12

**Authors:** Shang-Feng Tsai, Chia-Tien Hsu, Mu-Chi Chung, Ming-Ju Wu, Ya-Chin Huang, Cheng-Hsu Chen

**Affiliations:** 1 Department of Post-Baccalaureate Medicine, College of Medicine, National Chung Hsing University, Taichung 402202, Taiwan; acde0324@vghtc.gov.tw (S.-F.T.);; 2 Division of Nephrology, Department of Internal Medicine, Taichung Veterans General Hospital, Taichung 40705, Taiwan; 3 Department of Life Science, Tunghai University, Taichung 407224, Taiwan; 4 Ph.D. Program in Tissue Engineering and Regenerative Medicine, College of Medicine, National Chung Hsing University, Taichung 402202, Taiwan

**Keywords:** renal biopsy, pre-admission test (PAT), patient flow, admission periods, waiting time, quality improvement (QI)

## Abstract

**Background:** Admission for renal biopsy is considered the gold standard for diagnosing kidney disease. However, prolonged waiting times for admission can lead to delayed diagnosis. Despite this issue, there are currently no studies demonstrating how to improve the efficiency of renal biopsy procedures. **Methods:** We initiated a quality improvement project to implement pre-admission testing (PAT) for renal biopsy from 2016 to 2024 (until 15 April). Our evaluation focused on waiting times for admission, length of admission periods, hospitalization expenses, percentage of cases with no renal biopsy performed, incidence of severe bleeding due to renal biopsy, and percentage of cases with adequate tissue samples obtained. Additionally, we highlighted the time periods during the outbreak of SARS-CoV-2. **Results:** The highest annual case number was observed in time period 1 (168.3/year). Following the outbreak of SARS-CoV-2, there was a notable decrease in case numbers during time period 2 (119.8), which then increased to 143.0 in time period 3 (post-SARS-CoV-2 era). The mean waiting time was 13.72 ± 40.30 days for time period 1 and 10.00 ± 47.80 days for time period 2, without statistical significance. Following the implementation of PAT, patients now only need to wait approximately 0.76 days for admission, representing a significant reduction in waiting time. Subsequently, following the implementation of PAT, the waiting time decreased significantly to 2.09 ± 2.65 days. Additionally, hospitalization expenses per patient significantly decreased from approximately USD 69.62 ± 97.09 to USD 41.66 ± 52.82. The percentage of missed biopsy is significantly low (*p* < 0.001). Severe bleeding events (indicated as embolization and blood transfusion) were consistent across the three time periods (*p* = 0.617). **Conclusions:** The implementation of PAT can improve the pre-admission process for renal biopsy, resulting in decreased waiting times, fewer missed appointments, shorter admission durations, and reduced hospitalization expenses. We propose implementing PAT for outpatient individuals awaiting in-hospital renal biopsy procedures to mitigate delayed diagnosis, reduce pre-admission waiting periods, and streamline admission processes, thereby enhancing overall patient care efficiency.

## 1. Background

Renal biopsy stands as the cornerstone therapeutic modality for renal diseases [1], encompassing a spectrum of etiologies spanning acute and chronic kidney pathologies [2], whether of unknown origin, primary or secondary to renal conditions, or involving native or allograft kidneys. Presently, no diagnostic modality rivals the comprehensive insights provided by renal biopsy, elucidating the underlying etiology of renal dysfunction and informing the intensity of therapeutic interventions in both acute and chronic settings. Literature suggests that the absence of renal biopsy may engender diagnostic and therapeutic delays in the management of renal disorders [3,4]. Nonetheless, its invasive nature renders renal biopsy less prevalent in clinical practice [1]. Additionally, effective patient communication regarding procedural indications and potential complications is imperative in the nephrology setting. In our previous investigation, we introduced a communication model for shared decision making to foster clarity between healthcare providers and patients regarding renal biopsy [5]. Notably, our institution has reported minimal bleeding complications in allograft kidney biopsies [6], supported by rigorous clinical audits ensuring biopsy tissue quality. These endeavors have culminated in our institution conducting the highest number of renal biopsies nationwide, exceeding 9000 procedures, with certified expertise recognized by the Symbol of National Quality (SNQ) in Taiwan.

Given the invasive nature of renal biopsy procedures, in our county, it is advisable for them to be performed with the patient admitted to the hospital. Nevertheless, despite our institutional capabilities, patients frequently encounter extended waiting times for admission, resulting in delayed diagnosis and treatment, as well as loss of follow-up. Outpatient department (OPD) settings present challenges in securing a definite date for renal biopsy due to limited bed availability. Consequently, the scheduled date for renal biopsy remains tentative for patients. On the designated day of the biopsy, every effort is made to secure a bed for post-biopsy observation. However, in the event of bed unavailability, the biopsy schedule usually experiences delays. Our institute (Taichung Veterans General Hospital) (TCVGH) is a prominent medical facility boasting a capacity of 1624 beds and employing approximately 5500 staff members. Situated in Taichung City, it serves as a vital healthcare hub for the region. On a daily basis, TCVGH accommodates over 7000 OPD visits, 160 emergency room (ER) visits, conducts more than 3700 surgeries, and admits approximately 5000 patients. Hence, optimizing the pre-admission process for renal biopsy assumes paramount importance to expedite the diagnosis and management of renal diseases.

A previous study suggested policies to reduce excessively long wait times to improve outcomes for patients awaiting treatment for psychosis [7]. Another study [8] found that longer pre-admission waiting times were linked to increased postoperative complications, longer hospital stays, and higher hospitalization costs in older Chinese patients undergoing hip fracture surgery. Within two years, a review article [9] identified eight approaches to reducing surgical wait times: targeted funding, i.e., publicly funded but privately delivered services, patient prioritization, same-day surgery and discharge, streamlined pre-admission processes, expanded roles for non-physicians, standardized treatment pathways, process improvement methodologies, and regular validation of wait lists. To date, there are no studies focusing on how to reduce waiting times for in-hospital renal biopsies.

Pre-Admission Testing (PAT) serves as a crucial step in ensuring optimal surgical outcomes through pre-screening patients [10,11]. This process not only aids in preparing both patients and staff, but also prioritizes patient safety as the paramount objective. PAT not only demonstrated efficacy in streamlining surgical workflows, but also reduce same-day cancellations [12]. However, its integration into the renal biopsy pathway remains underexplored in the existing literature. Thus, recognizing this gap, we have initiated a quality improvement (QI) initiative aimed at incorporating PAT into our renal biopsy protocol. Through this endeavor, we aspire to disseminate our insights globally, aiming to mitigate waiting times and enhance the admission process in regards to renal biopsy.

## 2. Material and Methods

### 2.1. Standard Renal Biopsy Procedure at TCVGH

In the clinical setting of TCVGH, percutaneous renal biopsy is a standard procedure requiring patient admission, followed by meticulous monitoring of vital signs and potential bleeding manifestations. Subsequently, patients undergo abdominal sonography the day after the biopsy to assess for any signs of hemorrhage. Conditional upon maintaining stable clinical parameters, patients meeting discharge criteria are eligible for release from medical care.

### 2.2. Inclusion and Exclusion Criteria

The study included all outpatient individuals who underwent sonography-guided percutaneous renal biopsy at TCVGH between 1 January 2016 and 15 April 2024. We excluded those who opted for the procedure after hospital admission. This exclusion was based on the understanding that PAT would not provide substantial benefits to this subgroup. Additionally, we excluded computed tomography-guided renal biopsy procedures, as they were performed by radiologists.

### 2.3. Study Periods

Given the impact of the SARS-CoV-2 outbreak (2019–2022) on hospital admissions, a comparison was made across three distinct time periods. The QI initiative aimed at enhancing the pre-admission process for renal biopsy was initiated on 1 January 2023, and subsequently, the impact of PAT for renal biopsy was evaluated from the same year onward. Therefore, period 1 (1 January 2016–31 December 2018) represented the pre-PAT era before the outbreak of SARS-CoV-2, period 2 (1 January 2019–31 December 2022) denoted the pre-PAT era during the outbreak of SARS-CoV-2, and period 3 (1 January 2019–15 April 2024) signified the post-PAT era following the outbreak.

### 2.4. Outcome Analysis

Our analysis will entail a comparison of case volumes, waiting times for admission (in days), length of admissions (in days), and the percentage of non-admissions (%) before and after the implementation of PAT. The percentage of cases not undergoing renal biopsy despite being scheduled for admission refers to instances where patients have made arrangements for admission for renal biopsy but encounter an unavailability of beds upon their arrival, leading them to revisit the outpatient department. Additionally, we will assess the percentage of severe bleeding incidents (embolization or blood transfusion due to post-biopsy kidney bleeding) and the quality of biopsied tissue (specifically, the number of glomeruli observed in light microscopy, with <10 glomeruli considered inadequate [13]) (%) before and after the implementation of PAT. Furthermore, given the onset of the SARS-CoV-2 pandemic, we will specifically highlight this timeframe for analysis. All methods were carried out in accordance with the relevant guidelines and regulations of TCVGH (Institutional Review Board number: CE222291B-1).

### 2.5. Statistical Analysis

Data are presented as mean ± SD. One-way analysis of variance (ANOVA) was employed to compare the means for continuous variables, while Fisher’s exact test was utilized to compare the prevalence for dichotomous variables. Statistical analyses were conducted using SPSS software version 22 (SPSS Inc., Chicago, IL, USA). A two-tailed *p*-value < 0.05 was considered statistically significant.

## 3. Results

### 3.1. Pre-Admission Testing (PTA) in TCVGH

**Figure 1:**
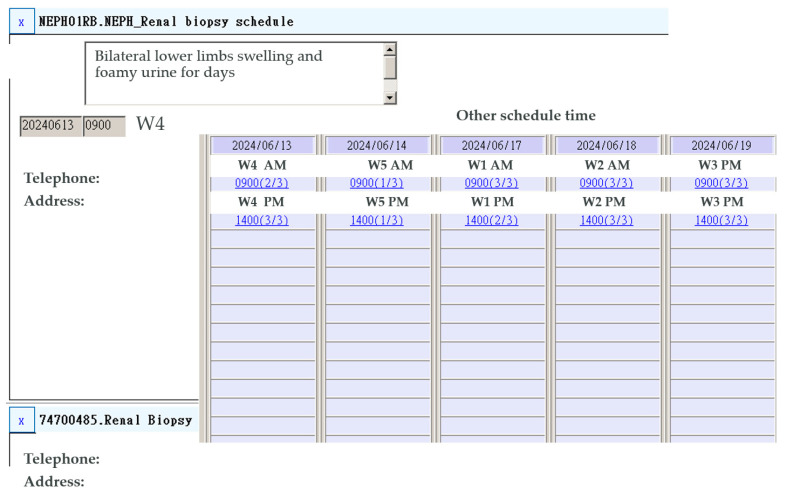
Scheduling renal biopsy admission during pre-admission testing in the outpatient department.

The QI initiative aimed at refining the pre-admission process for outpatient renal biopsy was initiated in 2023. Upon the decision for renal biopsy, a comprehensive set of diagnostic tests, including complete blood count, differential count, platelet count, prothrombin time, activated partial thromboplastin time, antinuclear antibody, double-stranded DNA, complement components C3 and C4, antineutrophil cytoplasmic antibodies, anti-glomerular basement membrane antibodies, hepatitis B surface antigen, anti-hepatitis B surface antibody, and anti-hepatitis C virus antibodies, are prescribed during the same outpatient visit. In other words, all the above tests were completed before the day of admission. Subsequently, patients are consulted regarding the scheduling of the renal biopsy procedure, which should occur within 7 days of the tests to ensure the safety of their anticoagulant status. All medications underwent a thorough review, including a collaborative effort between nephrologists and patients, ensuring the absence of any contraindications for renal biopsy. This comprehensive evaluation encompassed a range of medications, including antiplatelets, anticoagulants, non-steroidal anti-inflammatory agents, pentoxifylline, and persantine. Following this discussion, the date for the renal biopsy is arranged, and a bed for admission is reserved (see Figure 1). The Department of Medical Administration staff then inform the patients about their admission for renal biopsy and allocate a dedicated bed for their post-biopsy care.

**Figure 2:**
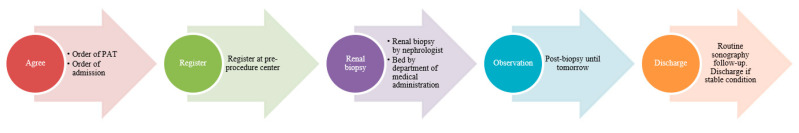
The patient flow for renal biopsy.

The procedural workflow of PTA for renal biopsy is outlined in Figure 2. On the scheduled date of the renal biopsy, patients are directed to the pre-procedure center at TCVGH. Subsequently, nephrologists are contacted by staff to perform the renal biopsy procedure as soon as possible. The patient undergoes renal biopsy in the dedicated renal biopsy room within the Nephrology Department, overseen by the nephrologist. Concurrently, the Department of Medical Administration ensures the allocation of an available bed for the patient’s post-biopsy care. Following the renal biopsy, patients are transferred to the ward for post-biopsy monitoring, which continues until the following day. On the second day post-biopsy, renal sonography is conducted for all patients to assess for any signs of bleeding. Upon confirmation of stable condition, patients are discharged from medical care. Usually, patients require a two-day hospital stay, spending one night, following renal biopsy, provided there are no severe complications detected during this improved process.

During the implementation of PAT, no major challenges were encountered. All nephrologists and patients expressed satisfaction with the QI initiative aimed at facilitating the renal biopsy process. The only minor challenge arose in the first month, when nephrologists were not familiar with the booking system in our Electronic Hospital Information System. However, by the second month of PAT implementation, all renal biopsy patients were smoothly processed through PAT.

### 3.2. Case Numbers of Renal Biopsy from Outpatient Individuals in TCVGH

In Table 1, the total and annual case numbers of renal biopsies at TCVGH are stratified according to whether they were conducted on native kidneys or allografts. The highest annual case number is observed in time period 1 (168.3/year). Following the outbreak of SARS-CoV-2, there was a notable decrease in annual case numbers during time period 2 (119.8). However, these numbers increased in time period 3 (143.0) during the post-COVID period.

### 3.3. Duration of Wait for Admission for Renal Biopsy (Days)

**Figure 3:**
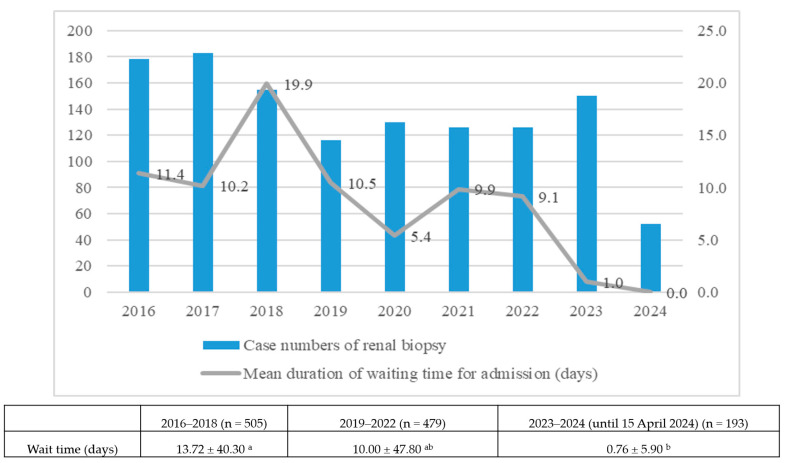
Duration of wait for admission for renal biopsy (days). ^a^: no statistical difference between time period 1 and 2. ^b^: no statistical difference between time period 2 and 3. Statistical difference between time period 1 and 3.

Figure 3 depicts the wait duration for admission, a crucial factor in determining the indication for renal biopsy. In time period 1, patients experienced wait times of approximately 11.4, 10.2, and 19.9 days for admission in years 2016, 2017, and 2018, respectively. However, during the SARS-CoV-2 outbreak, patients faced shorter waiting periods, with waits of 5.4 days in 2020, 9.9 days in 2021, and 9.1 days in 2022. The mean waiting time is 13.72 ± 40.30 days for time period 1 and 10.00 ± 47.80 days for time period 2, without statistical significance. Following the implementation of PTA, patients only waited approximately 1.4 days for admission in 2023 and had no wait time in 2024, representing a significant reduction. The mean waiting time for time period 3 decreased to 0.76 days, showing statistical significance.

### 3.4. Admission Duration and Hospitalization Expense for Renal Biopsy

Table 1 displays the admission duration for renal biopsy. In time period 1, the admission duration averaged around 3.74 ± 6.20 days. This decreased to 3.49 ± 4.90 days in time period 2, although this was not statistically significant. Subsequently, following the implementation of PAT, there was a significant decrease to 2.09 ± 2.65 days. Similarly, hospitalization expenses significantly decreased after the implementation of PAT, from around USD 74.68 ± 123.66 to USD 41.66 ± 52.82 per patient.

### 3.5. Percentage of Cases Not Undergoing Renal Biopsy despite Being Scheduled for Admission

**Figure 4:**
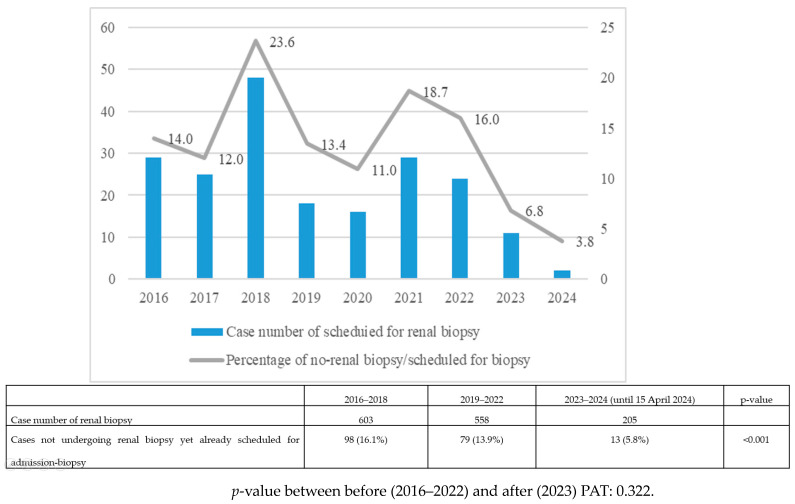
Percentage of cases not undergoing renal biopsy despite being scheduled for admission.

Figure 4 illustrates the percentage of cases not undergoing renal biopsy despite being scheduled for admission. In 2018, 23.6% of patients were unable to undergo admission for renal biopsy despite indications and scheduling. During the SARS-CoV-2 outbreak, this percentage decreased to 13.4% in 2019 and 11.0% in 2020. However, there was a subsequent increase, with percentages rising to 18.7% in 2021 and 16% in 2022. Following the implementation of PAT, the percentage notably decreased to 5.8% (*p* < 0.001).

### 3.6. The Impact of PAT on the Quality of Renal Biopsy and Bleeding

Table 2 displays the numbers of glomeruli observed under light microscopy, as well as severe bleeding (embolization or blood transfusion due to renal biopsy), across three time periods. Glomerular numbers between the time periods were as follows: 10.07 ± 6.00 in time period 1, 10.23 ± 5.54 in time period 2, and 11.83 ± 6.80 in time period 3. Glomerular numbers are comparable in time periods 1 and 2, but significantly higher in time period 3. Regarding inadequate tissue, the percentage also decreased after the implementation of PAT, dropping from around 50.5% to 38.1%.

Severe bleeding events, including embolization and blood transfusion, are presented in Table 2. During the study period, the percentages of embolization or blood transfusion were consistent across the three time periods (*p* = 0.617).

## 4. Discussion

Our study pioneers the utilization of PAT in non-surgical procedures, focusing particularly on renal biopsy. Through the standardization of admission processes, we can optimize patient flow, resulting in reduced waiting times and admission durations. Furthermore, we expect a decrease in the rate of loss to follow-up cases.

Improving admission and discharge processes can accelerate patient flow. The hospitalization process comprises three main stages: admission, an inpatient period, and the final stage involving discharge. Delays in discharge are noted to be multifactorial, encompassing facets such as the efficient delivery of medical care, the availability of resources, and the internal operational frameworks of the institution [14]. Typically, the greatest variation occurs in the number of discharges, emphasizing the importance of beginning efforts to reduce variation with the discharge process rather than the admission process [15]. Therefore, much effort has been directed towards discharge planning, which should ideally commence from the time of admission [16]. However, it is crucial to acknowledge that the diversity among causes of admission, disease etiologies, healthcare facilities, and their respective teams entails unique challenges, pressures, concerns, and priorities for each. From this study, we observed that improving patient flow (reduced wait time for admission and shortened admission periods) depends more on the efficiency of the admission process rather than on discharge planning. The reasons for this are as follows: Firstly, renal biopsy falls under elective admissions. In other words, at least in our institution, the Medical Administration staff prioritize allocating beds to patients who are critically ill or require emergency surgery. Therefore, there is a need to educate them about the urgency of renal disease diagnosis. Secondly, we had already implemented shared decision making on renal biopsy between patients and clinicians to enhance the pre-admission process, as per findings from our previous study [5]. Thirdly, renal biopsy is a relatively simple and standardized procedure compared to others. Additionally, we implemented direct observation of procedural skills to monitor the procedural skills of the nephrologists. Consequently, we observed a lower risk of severe bleeding for renal biopsy, as indicated in our previous report [6]. In summary, the post-admission processes for renal biopsy were typically predictable. Based on the above reasons, we believe that the enhancement of patient flow for renal biopsy should rely more on the admission process rather than on discharge planning.

After implementing PAT, in addition to reducing the rate of no renal biopsies, we also observed improvements in the admission process in our study, resulting in significantly shortened admission durations (from more than 3 days to 2 days) and reduced hospitalization experiences (saving around USD 28 per patient). This change occurred because before PAT, patients typically underwent admission on the 1st day for blood tests, followed by renal biopsy on the 2nd day, and discharge on the 3rd day. With PAT, patients now receive blood tests at outpatient services within one week of admission, undergo renal biopsy on the 1st day, and are discharged on the 2nd day. Therefore, PAT has also contributed to improved duration periods.

Based on a previous study [9], there are eight identified approaches with consistent positive evidence of effectiveness: targeted funding [17], i.e., publicly funded but privately delivered services [18], patient prioritization [19], same-day surgery and discharge [20], streamlined pre-admission processes [20], expanded roles for non-physicians [21], standardized treatment pathways [21], process improvement methodologies [22], and regular validation of wait lists [23]. Our QI initiative can enhance patient prioritization (confirmation of bed availability for renal biopsy by administrative staff), facilitate same-day renal biopsy due to PAT, streamline pre-admission processes (PAT and registration at pre-procedure), implement process improvement methodologies (as shown in Figure 2), and ensure regular validation of wait lists. Therefore, our QI initiative can undoubtedly shorten wait times and admission periods. Currently, there are no published studies investigating the admission process for in-hospital renal biopsy. We are the first to share our experience in this regard.

The impact of SARS-CoV-2 is also evident in this study. Because many surgeries and unnecessary admissions were canceled, more beds became available for patients requiring renal biopsy. Even though the outbreak of SARS-CoV-2 in Taiwan was not as severe as that in other countries [24,25,26], patients were still hesitant about admission for renal biopsy. The number of renal biopsies per year decreased from 168.3 to 118.0 due to the outbreak. However, during the outbreak (time period 2), patient wait time for admission was not as long as that in time period 1 (13.72 vs. 10.00 days). The percentage of missed renal biopsies also decreased (16.1% vs. 13.9%). In time period 3 (after the implementation of PAT and control of SRAS-CoV-2), the number of cases remained significantly increased (119.8 vs. 143.0), and the wait time for admission was further reduced (10.00 vs. 0.76 days), as was the percentage of missed renal biopsies (13.9% vs. 5.8%) (*p* < 0.001). In time period 3, despite the post-COVID rebound effect and increased admissions (such as for acute myocardial infarction [27]), we were able to shorten the wait time and admission periods for renal biopsy due to the QI initiative implemented to control SARS-CoV-2.

In addition to SARS-CoV-2, there may still be some potential confounding factors that could have influenced the outcomes, such as changes in hospital policies, increased manpower in nephrology, or patient demographics. However, during our study period, there were no changes in hospital policy (except for this QI initiative on renal biopsy), nephrology staffing, or patient demographics.

This study has a limitation. We lacked patient-reported outcomes, such as satisfaction or quality of life measures. Although we anticipated that reduced wait times and admission periods would lead to increased patient satisfaction, we did not directly measure these outcomes.

## 5. Conclusions

In conclusion, this study underscores the effectiveness of PAT in improving the pre-admission process for renal biopsy, resulting in decreased wait times, fewer missed appointments, shorter admission durations, and reduced hospitalization expenses. Additionally, PAT is associated with obtaining fewer inadequate tissue samples. We propose implementing PAT for outpatient individuals awaiting in-hospital renal biopsy procedures to mitigate delayed diagnosis, reduce pre-admission wait periods, and streamline admission processes, thereby enhancing overall patient care efficiency.

## Figures and Tables

**Table 1:** Renal biopsy case numbers for outpatient individuals, admission durations, and hospitalization expenses after 2016.

	2016–2018	2019–2022	2023–2024 (until 15 April)	*p*-Value
Graft kidney	212 (42.0%)	114 (23.8%)	34 (17.5%)	<0.001
Native kidney	293 (58%)	365 (76.2%)	159 (82%)	<0.001
Total case numbers	505.0	479.0	193	
Annual case numbers (/year)	168.3	119.8	143.0	
Admission duration (days) (mean ± SD)	3.74 ± 6.20 ^a^	3.49 ± 4.90 ^a^	2.09 ± 2.65 ^b^	
Hospitalization expenses covered by health insurance (USD/patient)	74.68 ± 123.66 ^a^	69.62 ± 97.09 ^a^	41.66 ± 52.82 ^b^	

^a^: no statistical difference between time period 1 and 2. ^b^: statistical difference between time period 1/2 and 3.

**Table 2:** Percentage of inadequate samples (number of glomeruli under light microscopy < 10) and severe bleeding (embolization and blood transfusion) due to biopsy.

	2016–2018 (n = 505)	2019–2022 (n = 479)	2023–2024 (until 15 April 2024) (n = 193)	*p*-Value
Tissue adequacy of renal biopsy				
Numbers of glomeruli	10.07 ± 6.00 ^a^	10.23 ± 5.54 ^a^	11.83 ± 6.80 ^b^	
Glomeruli under light microscopy < 10	265 (52.5%)	242 (50.5%)	71 (36.6%)	0.001
Severe bleeding complication				
Embolization due to post-biopsy bleeding	0	0	0	1
Blood transfusion due to post-biopsy bleeding	3	5	1	0.617

^a^: no statistical difference between time period 1 and 2. ^b^: statistical difference between time period 1/2 and 3.

## Data Availability

All data are contained in the paper.

## References

[1] Anders H.J., Huber T.B., Isermann B., Schiffer M. (2018). CKD in diabetes: Diabetic kidney disease versus nondiabetic kidney disease. Nat. Rev. Nephrol..

[2] Najafian B., Lusco M.A., Alpers C.E., Fogo A.B. (2022). Approach to Kidney Biopsy: Core Curriculum 2022. Am. J. Kidney Dis..

[3] Cohen A.H., Nast C.C., Adler S.G., Kopple J.D. (1989). Clinical utility of kidney biopsies in the diagnosis and management of renal disease. Am. J. Nephrol..

[4] Pfister M., Jakob S., Frey F.J., Niederer U., Schmidt M., Marti H.P. (1999). Judgment analysis in clinical nephrology. Am. J. Kidney Dis..

[5] Chen C.H., Hsu C.T., Wu M.J., Tsai S.F. (2022). Quality Improvement Initiatives in Renal Biopsy for Patient-Centered Communication by Shared Decision Making. Diagnostics.

[6] Tsai S.F., Chen C.H., Shu K.H., Cheng C.H., Yu T.M., Chuang Y.W., Huang S.T., Tsai J.L., Wu M.J. (2016). Current Safety of Renal Allograft Biopsy With Indication in Adult Recipients: An Observational Study. Medicine.

[7] Reichert A., Jacobs R. (2018). The impact of waiting time on patient outcomes: Evidence from early intervention in psychosis services in England. Health Econ..

[8] Chen X., Liao Z., Shen Y., Dong B., Hou L., Hao Q. (2021). The Relationship between Pre-Admission Waiting Time and the Surgical Outcomes after Hip Fracture Operation in the Elderly. J. Nutr. Health Aging.

[9] Stafinski T., Nagase F.N.I., Brindle M.E., White J., Young A., Beesoon S., Cleary S., Menon D. (2022). Reducing wait times to surgery—An international review. J. Hosp. Manag. Health Policy.

[10] Carel R.S., Kahan E., Hart J., Panush N. (1986). Preadmission testing in elective surgery—Improving the process. Isr. J. Med. Sci..

[11] Pierce D., Salinkar J., Yoon S.W., Khasawneh M.T. (2010). Pre-Admission Testing (PAT) Process Flow Optimization and Patient Chart Standardization for Elective Surgeries. https://www.researchgate.net/publication/290034437_Pre-Admission_testing_PAT_process_flow_optimization_and_patient_chart_standardization_for_elective_surgeries.

[12] CBS TEAM Focused Pre-Admission Testing Improvement Processes to Reduce Same-Day Cancellations. https://cbsteam.com/case-study/focused-pre-admission-testing-improvement-processes-to-reduce-same-day-cancellations/.

[13] Gerth J., Busch M., Illner N., Traut M., Gröne H.J., Wolf G. (2010). Are tissue samples from two different anatomical areas of the kidney necessary for adequate diagnosis?. Clin. Nephrol..

[14] Hunter M., Peters S., Khumalo N., Davies M.A. (2024). Analysis of patient flow and barriers to timely discharge from general medical wards at a tertiary academic hospital in Cape Town, South Africa. BMC Health Serv. Res..

[15] Cook J.L.E., Fioratou E., Davey P., Urquhart L. (2022). Improving patient understanding on discharge from the short stay unit: An integrated human factors and quality improvement approach. BMJ Open Qual..

[16] Zoucha J., Hull M., Keniston A., Mastalerz K., Quinn R., Tsai A., Berman J., Lyden J., Stella S.A., Echaniz M. (2018). Barriers to Early Hospital Discharge: A Cross-Sectional Study at Five Academic Hospitals. J. Hosp. Med..

[17] Queensland Health (2017). Guideline of Operating Theatre Efficiency.

[18] Wide Bay Hospital and Health Service (2018). The Wide Bay Wave.

[19] Raymond Spencer, on behalf of the Central Adelaide Local Health Network (CALHN) (2015). Central Adelaide Local Health Network Annual Report 2014–2015.

[20] Columbia B., Interior Health (2013). Interior Health Capital Strategy 2013–2023. https://www.interiorhealth.ca/sites/default/files/PDFS/ih-capital-strategy-2013-2023.pdf.

[21] Brown R., Grehan P., Brennan M., Carter D., Brady A., Moore E., Teeling S.P., Ward M., Eaton D. (2019). Using Lean Six Sigma to improve rates of day of surgery admission in a national thoracic surgery department. Int. J. Qual. Health Care.

[22] Valsangkar N.P., Eppstein A.C., Lawson R.A., Taylor A.N. (2017). Effect of Lean Processes on Surgical Wait Times and Efficiency in a Tertiary Care Veterans Affairs Medical Center. JAMA Surg..

[23] Kreindler S.A. (2010). Policy strategies to reduce waits for elective care: A synthesis of international evidence. Br. Med. Bull..

[24] Lai C.C., Lee P.I., Hsueh P.R. (2023). How Taiwan has responded to COVID-19 and how COVID-19 has affected Taiwan, 2020–2022. J. Microbiol. Immunol. Infect..

[25] Wu Y.H., Nordling T.E.M. (2024). Taiwan ended third COVID-19 community outbreak as forecasted. Sci. Rep..

[26] Chen Y.H., Fang C.T. (2024). Achieving COVID-19 zero without lockdown, January 2020 to March 2022: The Taiwan model explained. J. Formos. Med. Assoc..

[27] Fardman A., Oren D., Berkovitch A., Segev A., Levy Y., Beigel R., Matetzky S. (2020). Post COVID-19 Acute Myocardial Infarction Rebound. Can. J. Cardiol..

